# Electrospinning of Polystyrene/Polyhydroxybutyrate Nanofibers Doped with Porphyrin and Graphene for Chemiresistor Gas Sensors

**DOI:** 10.3390/nano9020280

**Published:** 2019-02-17

**Authors:** Joshua Avossa, Roberto Paolesse, Corrado Di Natale, Emiliano Zampetti, Giovanni Bertoni, Fabrizio De Cesare, Giuseppe Scarascia-Mugnozza, Antonella Macagnano

**Affiliations:** 1 Institute of Atmospheric Pollution Research–National Research Council (IIA-CNR), Research Area of Rome 1, Via Salaria km 29.300, 00016 Monterotondo, Italy; joshua.avossa@iia.cnr.it (J.A.); paolesse@uniroma2.it (R.P.); dinatale@uniroma2.it (C.D.N.); e.zampetti@iia.cnr.it (E.Z.); decesare@unitus.it (F.D.C.); 2 Department of Chemical Science and Technology, University of Tor Vergata, Via della Ricerca Scientifica 00133 Rome, Italy; 3 Department of Electronic Engineering, University of Tor Vergata, Via del Politecnico 1, 00133 Rome, Italy; 4 Institute of Materials for Electronics and Magnetism–National Research Council (IMEM-CNR), Parco Area delle Scienze 37/A, 43124 Parma, Italy; giovanni.bertoni@imem.cnr.it; 5 Department for Innovation in Biological, Agro-food and Forest Systems (DIBAF), Via S. Camillo de Lellis, 00100 Viterbo, Italy; gscaras@unitus.it

**Keywords:** electrospinning deposition, chemosensor, nanocomposite conductive polymers, polyhydroxibutyrate, polystyrene, H_2_TPP, VOCs selectivity, mesoporous graphene

## Abstract

Structural and functional properties of polymer composites based on carbon nanomaterials are so attractive that they have become a big challenge in chemical sensors investigation. In the present study, a thin nanofibrous layer, comprising two insulating polymers (polystyrene (PS) and polyhydroxibutyrate (PHB)), a known percentage of nanofillers of mesoporous graphitized carbon (MGC) and a free-base tetraphenylporphyrin, was deposited onto an Interdigitated Electrode (IDE) by electrospinning technology. The potentials of the working temperature to drive both the sensitivity and the selectivity of the chemical sensor were studied and described. The effects of the porphyrin combination with the composite graphene–polymer system appeared evident when nanofibrous layers, with and without porphyrin, were compared for their morphology and electrical and sensing parameters. Porphyrin fibers appeared smoother and thinner and were more resistive at lower temperature, but became much more conductive when temperature increased to 60–70 °C. Both adsorption and diffusion of chemicals seemed ruled by porphyrin according its combination inside the composite fiber, since the response rates dramatically increased (toluene and acetic acid). Finally, the opposite effect of the working temperature on the sensitivity of the porphyrin-doped fibers (i.e., increasing) and the porphyrin-free fibers (i.e., decreasing) seemed further confirmation of the key role of such a macromolecule in the VOC (volatile organic compound) adsorption.

## 1. Introduction

Chemical sensors are usually conceived as electronic devices comprising a sensing material, in charge of interacting with the target analyte, and a transducer, to transform such an interaction into an electric/optical signal [1]. Among the main drivers for the design of advanced chemical sensors, the key characteristics include sensitivity, selectivity, and rapid detection of target molecules. Over the last decade, the combination of nanostructured materials with many transducers has boosted the advances in this area, leading to significant enhancements in their sensing performance [2,3]. Consequently, together with a plethora of complex nanostructures, polymer nanocomposites have been designed and investigated as promising candidates for developing advanced materials for sensors. These joint materials benefit from the synergy between filler nanoparticles and polymer chains: they are both on similar length scales and with a very large interfacial surface area when compared to the volume of the material [4]. On the other hand, polymers are one of the most extensively exploited classes of materials due to the great variety of available chemical moieties with their relatively low cost, easy processing, and potential for designing and fabricating recycled and sustainable materials for sensors of the last generation [5]. More specifically, the development of polymeric composites based on carbon nanomaterials, such as carbon nanotubes (CNTs) and graphene (G), has been given a great deal of attention as a path to achieve new sensing materials with new structural (e.g., mechanical stability) and functional properties, likely better performing than pure components. Among the remarkable features of these two carbon allotropes to be used to design a chemical sensor, there are high electrical conductivity and large surface area [6]. Both nanomaterials have the same honeycomb lattice with sp^2^-hybridized carbon atoms, but with a two-dimensional sheet in graphene and its rolling up in one (SWNT, single-wall nanotubes) or more concentric tubes (MWNT, multi-wall nanotubes). Chemiresistors based on carbon-polymer combinations comprise features such as great stability, improvement of lifetime, tunable selectivity, ability to work at room temperature, good reversibility and reproducibility, low power consumption and cost effectiveness [7]. Generally, in these systems, current passes through continuous pathways of the conductive carbon particles between the parallel electrodes of the transducers. The sorption of a chemical vapor can cause softening/swelling of the polymer film, breaking some of the continuous pathways and increasing the resistance of the composite. Therefore, polymers can be designed to be more or less selective to different classes of VOCs, taking into account their solvation parameters according to vapor solubility (linear solvation energy relationships (LSER)) [8]. Obviously, much attention has been paid to properly modifying both polymers (functionalization of polymer chains) and polymer films (layer structure) to be simultaneously more selective to defined VOCs and optimized to host carbon nanofillers [9,10,11]. Therefore, chemiresistors based on Nafion–CNT [12] and poly(2,5-dimethylaniline)–CNT [13] have revealed intriguing performances in measuring air humidity and acid vapors, respectively. More recently, many nanocomposite polymer–graphene materials have been investigated and used successfully as sensors for industrial chemical reagents, drugs and explosives [14,15], thus highlighting comparable [16], and in some studies even better performances [17], than those of CNT-based sensors. This aspect sounds attractive since graphene is synthesized according to lower cost procedures and it is a material with the highest electrical conductivity known at room temperature (6000 Sc m^−1^) [18], a huge surface area (2.63 × 10^3^ m^2^ g^−1^) and a complete impermeability to any gases [19]. The efficiency of graphene–polymer composite seems to be related to the molecular-level dispersion of graphene [20] that commonly occurs by using a proper surfactant and/or selecting a polymer matrix that, through π−π stacking or hydrophobic (van der Waals) interactions, preserve the intrinsic electronic properties of graphene and allow a nanofiller homogeneous distribution [21]. Regarding a graphene hosting layer framework, thin and porous films, facilitating gas/VOCs diffusion, are preferred to get fast sensor responses and to avoid layer poisoning (hysteresis effects). A very porous layer with a controlled distribution of the nanofillers can be developed by electrospinning (ES) deposition. This is a technology able to produce advanced multifunctional polymer nanocomposites (2D and 3D micro- or nanofibrous layers) and has been conceived as one of the most promising strategies to design and fabricate highly sensitive nanocomposite films at low cost and with high production rates [22]. The process uses a high voltage to provide sufficient charges in the polymer solution such that a jet is ejected from the tip of a spinneret toward a grounded collector: the solvent evaporates on the path and the polymer nanofibers can be grown according to various arrangements and different morphologies. Thus, graphene nanoparticles can be added in the polymer solution for electrospinning, and nanofibers can be investigated as potential conductive material for chemical sensors. In a recent study, Avossa et al. [23] used the temperature to modulate the sensitivity of nanocomposite polymer nanofibers (polystyrene, polyhydroxibutyrate, mesoporous graphitized carbon (PS-PHB-MGC)) to gas and VOCs, making the sensor more selective to NO_2_ (LOD: 2 ppb, limit of detection) when operated at 80 °C, whereas VOCs adsorption decreases. 

A polymer doping agent is an alternative to the functionalization or substitution of polymer matrix for tuning the sensor selectivity. Different porphyrin species have been used to dope organic polymer. For instance, porphyrins combined with poly(2-phenyl-1,4-xylylene) have led to a huge variation in responses depending on the selected analytes (toluene, ethylacetate, ethanol, and propanone) [24]. Porphyrins have excellent sensing properties (their framework, peripheral substituents and the core that could be practically occupied by all metals of the periodic table) that make them an effective object of study and sensor applications of the last thirty years [25]. In the literature, there are several cases of porphyrins subjected to electrospun deposition in combination with electrospinnable polymers, so that their dispersion [26] and arrangement inside fibers as well as their photocatalytic [27] and sensing features [28,29] have been investigated extensively [30]. On the other hand, the best performances seem to be achieved when porphyrin occupies the outer part of the fiber [31] or when polymer fibers are very porous. Electrospun polymer fibers with a ternary composite combination (porphyrin, graphene oxide and nylon) are also manufactured [32] to be investigated towards more advanced applications.

In this paper, we present the design and the creation of a nanofibrous conductive chemical sensor based on a quaternary combination of two insulating polymers (PS and PHB, named as PsB) doped with 5,10,15,20-tetraphenylporphyrin (H_2_TPP) and mesoporous graphene nanopowder. Porphyrin, due to its molecular structure, is expected to give a significant contribution to the sensing properties of the polymer composite fibers [23], by coordinating the planar surfaces of MGC and the phenyl rings of PS (effects on fiber morphology and structure) and “capturing” selectively volatile organic compounds (VOCs) (effects on sensor sensitivity and selectivity). The polymers were selected because they are versatile (widely used in many consumer products), eco-compatible, biodegradable (PHB), recyclable (PS) [33,34] and resistant to thermal excursions (thermoplastics). Additionally, they are both soluble in chloroform and insoluble in H_2_O, meaning that they could be deposited by a unique electrospun mixture with a single needle, and were expected to be stable to changes in environmental humidity. In this more complex fibrous matrix, a preliminary study on the dependence of the sensor selectivity on the temperature was also carried out and is described below.

## 2. Materials and Methods 

Mesoporous graphitized carbon nanopowder (MGC) (<500 nm; available surface area: 50–100 m^2^/g; average pore diameter: 137 Å), hexadecyltrimethylammonium bromide (CTAB) (~99%), polystyrene (PS) (Mw = 192,000 g/mol), chloroform (≥99%), toluene (≥99.8%), acetic acid (≥99%), and poly[(R)-3-hydroxybutyric] acid (PHB) (natural origin) were purchased from Sigma-Aldrich (Merck KGaA, Darmstadt, Germany). Ethanol (≥99.8%) was obtained from Fluka (Buchs, Switzerland). All chemicals were used without further purification. A 5,10,15,20-tetraphenylporphyrin (H_2_TPP) was prepared following literature protocol [35]. Standardized pure air (5.0) was purchased from Praxair-RIVOIRA (Rome, Italy) and stored in cylinders. 

Interdigitated Electrodes (IDEs), provided by Micrux Technologies (Oviedo, Spain), were fabricated on borosilicate substrate (IDE sizes: 10 mm × 6 mm × 0.75 mm; Pt/Ti electrodes, 120 pairs, 10 μm wide × 5 mm long × 150 nm thick, with 10 μm gap) and rinsed with soap and a “base piranha” mixture at 60 °C for ~15 min, (3:1, v/v, ammonia water and hydrogen peroxide water solution) and finally with Milli-Q water (~18 MΩ cm) before any use.

The electrospun dispersion was prepared by first solubilizing 450 mg of PS pellets into 9 mL of chloroform under magnetic stirring. After complete dissolution, 60 mg of PHB were added into the solution and mixed at 50 °C for 2 h. Then, 150 mg of CTAB and 1 mL of ethanol were poured into the system and mixed overnight at 50 °C under magnetic stirring. Suspensions composed of 1.3 mg of MGC with and without 15 mg of H_2_TPP were poured into 2 mL of the PS/PHB/CTAB solution and sonicated for at least 1 h.

The resulting polymer dispersions were loaded into glass syringes (1 cm long stainless steel and blunt tips) and connected to a syringe pump (Model KDS 200, KD Scientific). The fibers depositions were carried out in a home-made (IIA-CNR, Monterotondo, Rome, Italy) and ventilated clean box equipped at ambient condition. The electrospinning apparatus consisted of a high power AC-DC converter, a high voltage oscillator (100 V) driving a high voltage (ranging from 1 to 50 kV), a syringe pump and a rotating conductive pipe with a 45 mm diameter grounded collector. The fibrous layers were fabricated by applying ~6 kV DC voltage between the syringe tip and the collector (8 cm of distance), at a pump feeding rate of 700 μL h^−1^. Deposition time was fixed at 2 min to obtain a thin and adhering coverage of the surface (IDEs and SiO_2_ wafers and High Precision Quartz slices). UV-Vis spectrophotometer (UV-2600 Shimadzu, Kyoto, Japan) was used to collect UV spectra of the fibrous layer at the solid state. 

Optical micrographs were captured by a Leitz-Wetzlar (Metallux 708082, Wetzlar, Germany) microscope, for the evaluation of the quality coverage of the fibers deposited onto the IDE.

Fibers morphological analyses were carried out by means of micrographs from Scanning Electron Microscopy (SEM) and Transmission Electron Microscopy (TEM). The electrospun nanofibrous fabrics deposited on thin SiO_2_ wafers and sputter-coated with gold in a Balzers MED 010 unit were analyzed for SEM by a JEOL JSM 6010LA electron microscope (High Equipment Centre, University of Tuscia, Viterbo, Italy). Scanning transmission electron microscopy (STEM) images were acquired in annular dark field mode (ADF) on a JEOL JEM-2200FS microscope operated at 200 kV and with a spot size of 2 nm (IMEM-CNR, Parma, Italy). The H_2_TPP/PS-PHB-MGC fibers were deposited on a lacey carbon coated copper grid, to reduce electrostatic charging of the fibers under the electron beam. Chemical mapping of carbon and oxygen were obtained from energy dispersive X-ray spectroscopy (EDXS) using a Si-Li detector (JEOL JED-2400, Akishima, Tokyo, Japan). 

The resulting chemiresistors (IDEs + NFs, where NFs means nanofibers) were sealed in a measurement glass chamber (~100 mL volume) and connected to an electrometer (Keithley 6517, Solon, Ohio, USA) capable of measuring their electrical parameters and sending data to a PC (LabVIEW 2014 Software, National Instruments, Austin, TX, USA). The current, provided under dry and clean air, was recorded by applying potential values from −4.0 to 4.0 V in steps of 0.4 V at different temperatures (25, 40, 50, 60, and 70 °C). Current versus applied voltage values were used to calculate the resistance of the fibrous coated IDE and its correlation to the temperature, as well as a potential hysteresis of the material when it was electrically stressed. All batches of the chemiresistors fabricated on different dates but keeping the identical deposition parameters reported the same electrical features, confirming the reproducibility of the deposition technique. 

Dynamic sensor measurements were carried out at different working temperature (T_w_: 50, 60, and 70 °C, generated by a micro-heater placed below each sensing area of the IDE) using: (i) 4-channel MKS 247 managing up to four MKS mass flow controllers (MFC), set in the range 0–200 sccm (standard cubic centimeter per minutes); and (ii) Environics S4000 (Environics, Inc., Tolland, CT, USA) flow controller, containing three MFCs supplying three flow rates (up to 500, 250 and 2.5 sccm, respectively), managed by its own software. Pure air was used as the gas carrier and it was blended with increasing concentrations of vapors of water (H_2_O), acetic acid (AcAc) and toluene (Tol), respectively, obtained through air bubbling in customized borosilicate bubblers (Rolando Spaziani S.r.l., Nettuno, Rome, Italy). A total gas flow of 300 sccm passed through the measurement chamber, housing the IDEs. Each measurement was carried out after the complete recovery of the starting current (the baseline) under dry and clean air flow. IDE responses were calculated as ΔI/I_0_, where ΔI is the current variation and I_0_ is the current when the air flowed.

## 3. Results and Discussion

**Figure 1:**
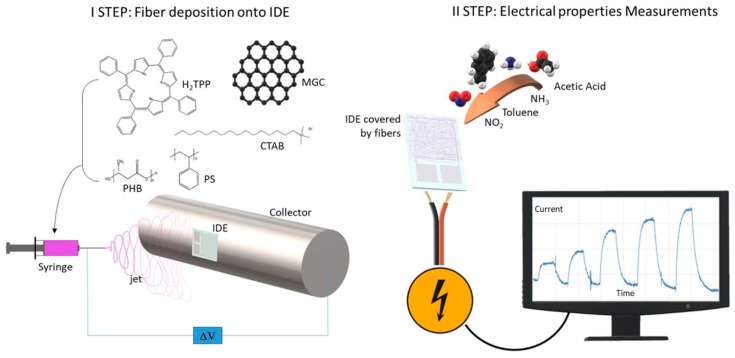
Sketch of electrospinning technique able to coat an IDE (interdigitated electrode) with composite nanofibers (**left**); and current measurements upon interaction of the chemosensor with gaseous molecules (**right**).

Electrospinning technology was used to create nanocomposite nanofibrous layers in a single step using a single needle. The depositions were easily carried out onto several substrates, specifically silicon dioxide thin slices (for fibers morphological, chemical and optical characterization) and customized borosilicate IDE transducers (for measuring electrical and sensing features of the thin nanofibrous coating). Each substrate, fixed onto the grounded rotating cylinder facing the needle tip, was able to collect the ejected fibers within the deposition cone (Figure 1). Fibers did not look aligned over the electrode, but arranged to form a porous network placed on the IDE surface. Despite the heterogeneity of the polymer suspensions, the electrospun jet streams occurred without discontinuity, so that fibers were collected for a few minutes.

**Figure 2:**
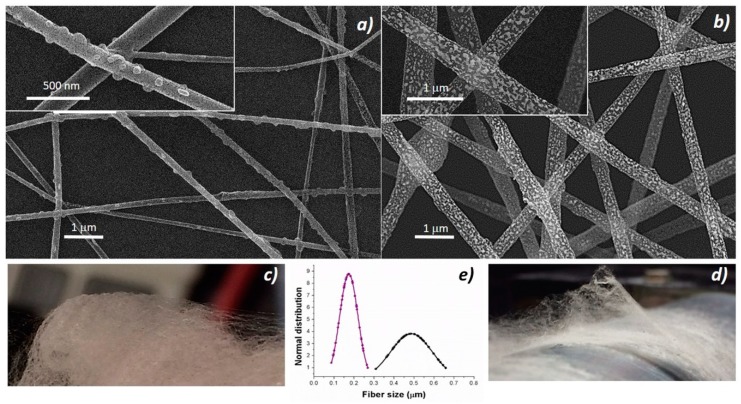
SEM micrographs of H_2_TPP-PsB-MGC (**a**) and PsB-MGC (**b**) [23] and their respective pictures placed under (**c**,**d**). Diameter distribution graph (**e**) of H_2_TPP-PsB-MGC (purple) (**a**) and PsB-MGC fibers (black) (**b**).

Shortly, the deposition process was generated by the application of a definite electrical field between the polymer suspension droplet at the metal nozzle and the grounded substrate placed at a distance. Due to the application of the electrical field, the polymer drop first changed shape (from spherical to conical) and then was elongated until the electrostatic forces exceeded the surface tension of the polymer suspension and forced the ejection of the liquid jet. Finally, dry and fine fibers were collected following the jet bending and stretching processes (by forces with opposing effects) [36], solvent evaporation and then splaying. The resulting fabrics appeared soft and cotton candy-like, and resulted easy to peel when electrospun processes were carried out for a longer time. Thus, to improve the fibers adhesion, it was necessary to thoroughly clean all the surfaces (base-piranha solution) and, following the deposition, incubate all the substrate at 60 °C under slight vacuum. The nanocomposite fabrics were pink (Figure 2c), turning to orange when the thickness increased but white/light gray (Figure 2d) if porphyrin-free. Nanocomposite fibers without porphyrin (PsB-MGC) previously investigated [23] appeared extremely rough on the surface and decorated with brighter islands, but fairly uniform in term of shape (cylindrical) and size (d: 550 ± 170 nm) (Figure 2b). The fibers combined with H_2_TPP (H_2_TPP-PsB-MGC) kept the same circular cross-sectional shape but appeared much smaller in size (d: 174 ± 50 nm) (Figure 2a). A possible reason for this phenomenon could be attributed to the polymer percentage decreasing in the final electrospun mixture [37,38] when porphyrin molecules were added. Furthermore, a higher voltage applied to the porphyrin mixture necessary to engage the electrospun process (V_H2TPP-PsB-MGC_ =≈ 6 kV; V_PsB-MGC_ = 2.9 kV) could also be responsible for the fiber diameter reduction when combined to a lower feed rate (i.e., 700 μL h^−1^ and 900 μL h^−1^ used for H_2_TPP- PsB-MGC and PsB-MGC suspensions, respectively) [39,40]. Porphyrin fibers looked smoother and had small spherical/elliptical bumps protruding from the whole the surface of the fibers (Figure 2a, inset). Therefore, the addition of the porphyrin to the composite system seemed to substantially change the morphology of the resulting fibers: in addition to dispersion forces, H_2_TPP could interact with PS and graphene by π−π interactions and with PHB by hydrogen bond. However, long, continuous and unbeaded fibers proved an appropriate combination of electrospinning set of parameters. 

**Figure 3:**
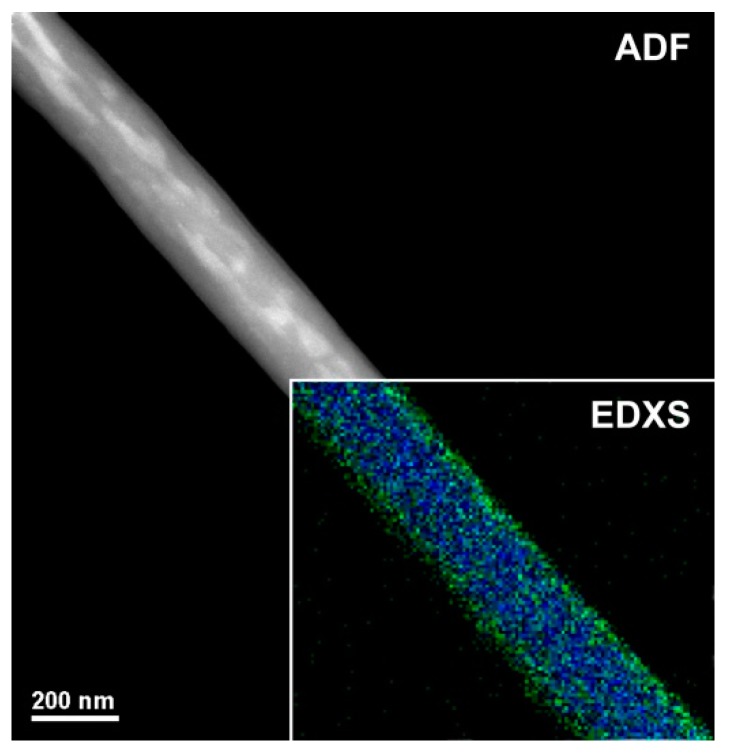
Annular dark field mode-scanning - transmission electron microscopy (ADFM-STEM) image of a porphyrin doped fiber. The inset shows the corresponding energy dispersive X-ray spectroscopy (EDXR) chemical map from carbon (blue) and oxygen (green).

Figure 3 shows a magnified TEM micrograph focusing on a single H_2_TPP- PsB-MGC fiber. The shape is regular but the heterogeneity of the fiber seems to be confirmed by areas with different contrast among the diverse nanoaggregates. For ADF-STEM imaging, the contrast (brightness/darkness) is approximately proportional to the square of the averaged atomic number projected in beam direction *z* and it depends linearly on the thickness [41] (in particular, the bigger is the atomic number, the brighter is the image). The bright regions inside the polymer/porphyrin fiber could be due to Br^−^, counterion to CTA^+^ (Cetyltrimethylammonium) in the surfactant. The cationic surfactant was used to decrease the aggregation of MGC particles and improve their solubility/stability in the polymer matrix. Thus, the brightest area (a higher scattering, i.e., higher intensity in the image) distributed along the inner part of the fiber is supposed to be consequently and indirectly related also to graphene dispersion. As concerns the distribution of both polymers (PS and PHB), the electrospun nanofibers were expected to result in a bulk matrix mainly composed of one of the two polymers hosting an approximately inhomogeneous dispersion of the second polymer, as a consequence of the poor polymer–polymer miscibility. According to the literature, when two polymers are soluble in the same solvent but incompatible with each other, they solidify in different domains [42] during fiber formation [43]. In the inset of Figure 3, the EDXS chemical map is shown, as obtained from C-K (blue) and O-K peaks (green). Oxygen looks to be more concentrated at the surface of the fiber, leading us to suppose a higher presence of PHB at the surface (carbonyl, hydroxyl and ether groups). On the other hand, the distribution of the porphyrin can hardly be highlighted by this technique since H_2_TPP is also substantially constituted by C atoms with the exception of the four N atoms in the core of the macrocycle (but having close atomic number); thus, the contrast is too low to recognize the porphyrin. A high affinity between PS and H_2_TPP (π−π stacking of the aromatic rings) is reported in literature where the homogeneous dispersion of the porphyrin in the surface and inside PS electrospun fibers is described using fluorescence microscopy (homogeneously red colored fibers) [28]. A weak π–π stacking could also occurs between graphene flakes surface and porphyrin molecules [44]. Therefore, porphyrin could be fairly dispersed among PS chains and MGC nanofillers in fiber inner part, and PHB arranged to the outermost part. 

**Figure 4:**
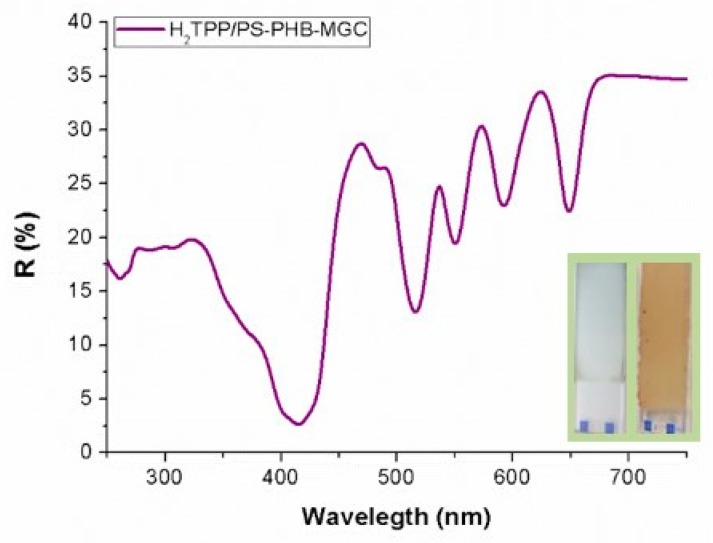
Diffuse reflectance ultraviolet-visible (DR-UV-Vis) spectrum of a H_2_TPP PsB-MGC thick fibrous layer (the orange one in inset). Inset shows also a porhyrin-free fibrous coating (the white-grey one).

The UV-Vis diffuse reflectance (R%) spectrum of a H_2_TPP-PsB-MGC fibrous layer (Figure 4) showed the characteristic features of the H_2_TPP chromophore, with the Soret (reflectance minimum about 2.5% at 415 nm) and Q bands well defined (VI: 516 nm, R: 13%; III: 550 nm, R: 19%; II: 591 nm, R: 23%; I: 648, R: 22%). Although the Soret band showed the expected broadening in the solid state, the absence of wavelength shifts seemed to indicate that the porphyrin could be well dispersed in the polymeric matrix. This hypothesis is also supported by the narrow Q bands.

**Figure 5:**
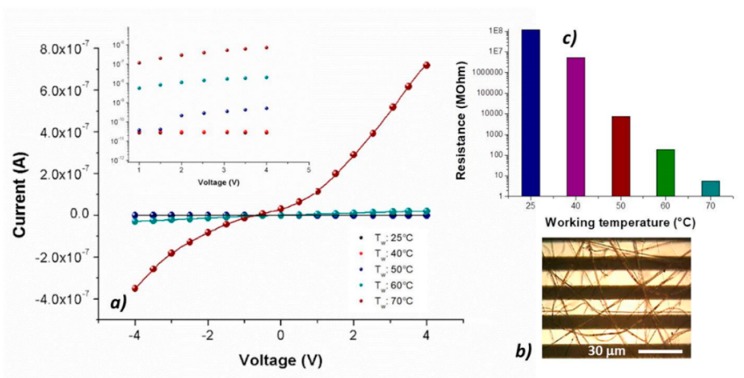
Current vs. voltage diagram at 25, 40, 50, 60 and 70 °C and 25, 40, 50 and 60 °C in the inset (**a**); optical image of the IDE covered by H_2_TPP-PsB-MGC fiber (**b**); and resistance values diagram at 25, 40, 50, 60 and 70 °C (**c**).

The nanocomposite fibrous layer, comprising many interfaces between each component. was expected to be an intriguing system for the development of chemical sensors, due to both the wide adsorption surface and the surface energy potentials involved [45]. Optical microscope pictures (Figure 5b) depicted a Pt-Ti microtransducer coated with 2 min-deposited fibers, which appeared optically transparent and with some small black MGC aggregates spread inside fibers, suggesting that the graphene distribution was not completely homogeneous but enclosed within the polymer wires. Increasing the deposition time, a thicker layer was obtained, but the adhesion resulted inhomogeneous and it was more easily peelable from the transducer. To measure the electrical parameters of the resulting chemiresistor, both at room and increasing temperatures up to 70 °C, each IDE, during the current vs. voltage measurements, was positioned onto a customized micro-heater fabricated on alumina substrate. The supplied voltage ranged between −4 V and +4 V. Current–voltage curves displayed a quasilinear relationship between the current changes and the imposed increasing voltage values. Indeed, both PS and PHB, being thermoplastics, can be heated to their melting point (T_M_PS_: 240 °C; T_M_PHB_: 175 °C), cooled, and reheated again without substantial degradation. The experimental melting point of porphyrin is also high enough (T_M_H2TPP_: ≥300 °C [46]) to allow the sensor to work properly in the established range. However, further heating involved an initial increase in current followed by a slow and irreversible increase in resistance, probably due to an irreversible arrangement of MGC inside fibers. For this reason, all experiments were carried out up to 70 °C. To investigate the contribution of the porphyrin to the chemiresistor electrical features, the same amount of graphene was used [23] to produce the fibers with and without porphyrin: indeed, upon the addition of porphyrin, MGC final mass percentage resulted about 1%. However, at room temperature (25 °C), porphyrin chemiresistor was more resistive (about 1.2 × 10^8^ MΩ) than the porphyrin-free one (about 3.4 × 10^6^ MΩ). The current inside fibers is supposed to occur by tunneling of electrons among MGC particles through a small insulating barrier (percolation theory). The electrical properties of such fibrous layers depend on both the quantity and the quality of the “texture” covering the electrodes. Therefore, the resulting measured electrical resistance is related to the individual fiber resistance (due to its dimension and shape), the fiber density (number of fibers per unit of surface area) and the electrode coverage. In porphyrin-fibers, nanofillers appeared distributed within the inner part of fibers, whereas the MGC aggregation inside PsB-MGC was supposed outer, then closer to the IDE metal-electrodes and with a smaller polymer barrier. A reason of this effect could be due to the arrangement of MGC inside fibers due to the porphyrin addition. Furthermore, it is known that conductivity can increase more than one order of magnitude when fiber diameter increases [47]. Porphyrin fibers were estimated to be much thinner than PsB-MGC one. On the other hand, thinner fibers are commonly preferred for sensor applications, since smaller-diameter wires are expected to have a faster response associated with a quicker diffusion of gas molecules through the fiber. Additionally, the collected porphyrin fibrous coatings showed a lower density of the PsB-MGC nanofibers over the electrodes (i.e., layer more porous, Figure 5b), although it increased with the same deposition time. This result could be a further reason to explain a lower conductivity of H_2_TPP fibrous chemiresistor. The electrical signals at 25 and 40 °C were noisy, thus the porphyrin chemiresistor did not seem to work properly. Conversely, increasing the electrode working temperature, current increased considerably, especially when temperature value was set at 70 °C. Consequently, the signal to noise ratio increased too, and the baseline looked more stable.

**Figure 6:**
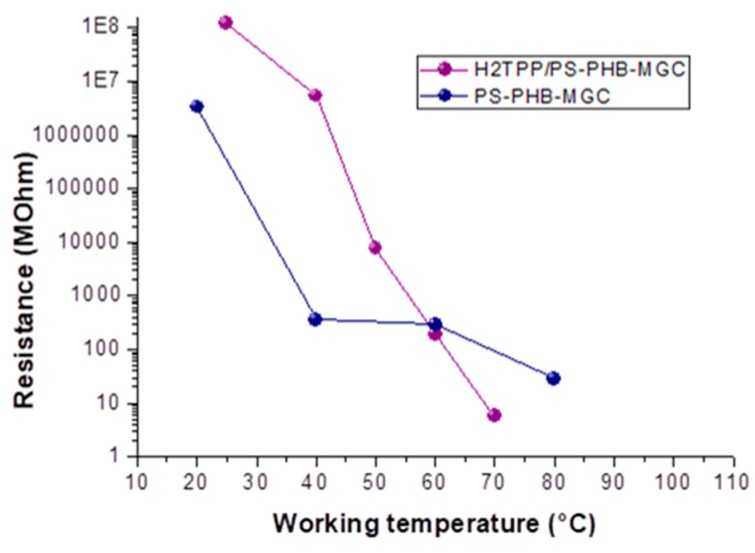
Resistance vs. working temperature for H_2_TPP-PsB-MGC (purple) and PsB-MGC (blue) fibers.

When the sensor was heated from 25 to 40 °C, the electrical resistance changed by two orders of magnitude (from 10^8^ to 10^6^ MΩ) (Figure 5c and inset). The exponential decreasing of the resistance values to the increasing 10 °C steps has been reported in the semi-logarithm bar-plot of Figure 5c, where resistance changed from ≈7 × 10^3^ to ≈5 MΩ, going from 50 to 70 °C. Such a non-linear dependence of conductivity on the heating apparently confirms the prevalence of the tunneling current (depending on the small dielectric barriers (insulating polymer) between the particles [48]) in comparison with the contact one [49,50], which usually should dominate in highly filled composites in contact with each other. The tunnel contribution is described by Equation (1):

(1) $$\rho_{T}=e^{\left(\frac{\pi w}{2}\sqrt{\frac{2mV_{0}}{{\left(\frac{h}{2\pi }\right)}^{2}}}\right)}$$

where $\rho_{T}$ is the tunnel resistivity, m is the electron rest mass, h is Planck’s constant, V_0_ is the height of the barrier, and w is its width [51]. Commonly, in a polymer nanocomposite matrix, the gap among nanofillers tends to increase with temperature due to the polymer phase volume expansion (i.e., polymer crystalline phase melting [52]) or the amorphous phase softening [53] during melting, resulting in a resistivity rise of several decades. In the fibrous chemiresistors here described, with and without porphyrin [23], the different results could be explained by the phenyl group rotation in polystyrene (polymer backbone chain reorientation [54]), which could favor strong π–π interactions existing between aromatic organic molecules and the basal plane of MGC. Such a rearrangement could be responsible for the connectivity improvement of the conductive network of the nanofillers, inducing the enhancement of the electrical conductivity [55]. The interfacial force between graphene and PS could be enhanced by surface modification, which reduced the interfacial thermal resistance and dispersed graphene more uniformly [56]. A significant contribution to the MGC rearrangement inside fibers could also be generated by the aromatic planes of H_2_TPP facing graphene surfaces. Indeed, comparing both the fibrous layers resistance values, H_2_TPP-PsB-MGC became less resistive than PsB-MGC, when the working temperature reached and went over 60 °C (Figure 6), notwithstanding the disadvantaged parameters listed above of the porphyrin layers to be conductive. More specifically, its resistance value reached ≈5.7 MΩ versus ≈81.6 MΩ of PsB-MGC measured at the same temperature (T_w_: 70 °C) in dry and clean air. Furthermore, the steep slope of H_2_TPP-PsB-MGC curve (Figure 6) could also be affected by the temperature effects on charge transport among porphyrin that is strongly temperature dependent [57]. It means that at lower temperature H_2_TPP could work as barrier while at higher temperature it should promote conduction. 

**Figure 7:**
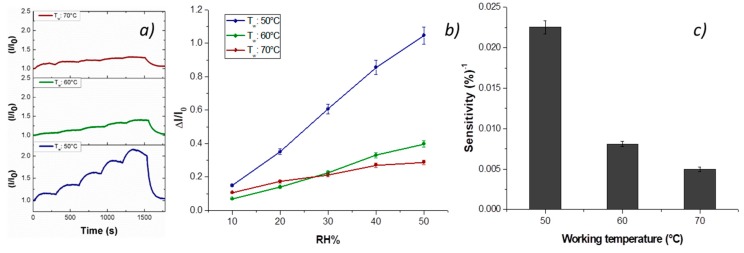
Normalized current (I/I_0_) versus time during water vapor measurement (10%, 20%, 30%, 40% and 50% RH) at 70, 60 and 50 °C (**a**); ΔI/I_0_–RH percentage diagram (response curves) (**b**); and sensitivity of the H_2_TPP-PsB-MGC electrode at the different working temperatures (**c**).

Vapor measurements were carried out according to a dynamic mode: the sensor was exposed to a gaseous stream of molecules, the content of which was ruled by blending a stream of pure air with a second stream saturated with the vapor to be analyzed. Thus, the fibrous chemosensor was firstly deployed to increasing percentages of water vapors, ranging 0–50% with increments of 10%, and with working temperature values set at 40, 50 and 60 °C. Both transient responses and response curves are depicted in Figure 7a,b. The current, reported as I/I_0_ (I: the current value; I_0_: starting current value under clean air) linearly increased when humidity percentage increased, too. A reason for this positive trend was the presence of the cationic surfactant inside the fibers that facilitated the dispersion of the carbon nanostructures [58]. Another reason could be related to the structure of the nanofillers, having here a mesoporous configuration, capable of easily entrapping water molecules and then interacting by the oxygen atoms making part of the framework of each MGC sheet [23]. When the chemosensor (T_w_: 50 °C) was exposed to 50% relative humidity, it became seven times more conductive than the dried one. An inverse relationship occurred when working temperature increased: under 50% RH (relative humidity), the sensor became six (60 °C) and three times (70 °C) more conductive than under dry air, therefore generally the sensor responses to water vapors decreased with temperature. The related sensitivity values, defined as the change of measured signal per analyte concentration unit, i.e., the slope of the sensor responses graph [59] (Figure 7c), showed a decrease of 64% and 78% when the sensor worked, respectively, at 60 and 70 °C. Sensitivity is a key parameter in sensor design, because it represents an index related to the sensor ability to “capture” an analyte. In fact, such a fibrous layer was designed to be scarcely affine to water molecules, having two hydrophobic polymers (PS and PHB) and planar structures of H_2_TPP and MGC preferring π–π interactions (despite H-bonds due to MGC structural defects and a cationic surfactant water soluble). Further, they were insoluble in H_2_O, meaning that the resulting fibers could be exposed to a wide range of relative humidity percentages without undergoing structural changes. The lowering of the affinity index to vapor due to the heating should be presumably caused by the decrease of the vapor molecules diffusion mainly caused by the backbone polymer chains motion [60,61]. In addition, in agreement with the kinetic theory of matter, the sorbed molecules (H-bonds and Van der Waals forces), gaining kinetic energy when heated, were able to “fly” out of the binding site. Indeed, furnishing the sufficient kinetic energy to desorb the “captured” material is the usual strategy to restore/regenerate an adsorbent [62]. A very similar behavior is reported for PsB-MGC nanosensors [23].

**Figure 8:**
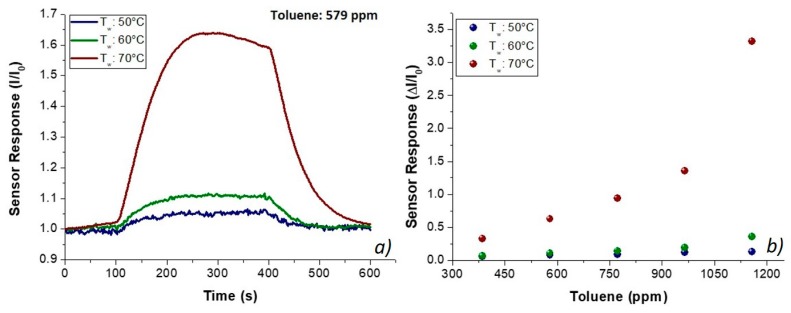
Transient responses (I/I_0_ versus time) upon injection of 579 ppm of toluene in dry air (**a**); and response curves (ΔI/I_0_) to different toluene concentrations at increasing temperature values (50, 60 and 70 °C) (**b**).

**Figure 9:**
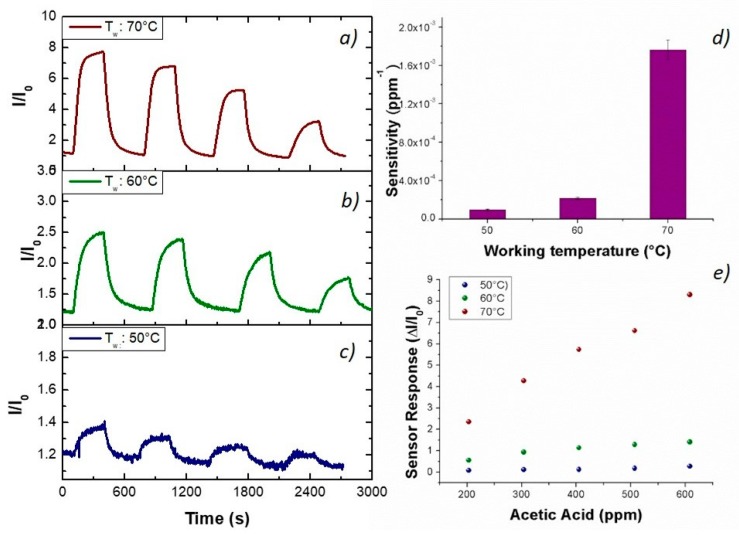
Transient responses (I/I_0_ versus time) upon injection of different concentration of acetic acid in dry air at: 70 °C (**a**); 60 °C (**b**); and 50 °C (**c**); sensitivity dependence on the working temperature (**d**); and response curves (ΔI/I_0_) to increasing acetic acid concentrations and increasing temperature (**e**).

To investigate a potential role of the porphyrin as a selective sensing agent, the fibrous chemosensor was deployed to toluene and acetic acid vapors, the two chemicals that, among the VOC chemical classes previously tested, reported the lowest and the highest, respectively, affinity to PsB-MGC fibers. Additionally, when working temperature increased, the PsB-MGC sensitivity values decreased greatly, making that sensor suitable to work at room (or close to room) temperature for VOC detection. Furthermore, the interest in testing both the chemicals is reinforced by the fact they have proved to be common organic indoor air pollutants [63] and extremely toxic by inhalation [64,65]. Finally, both acetic acid and toluene, being solvents of PHB and PS, respectively, were expected to be able to easily penetrate inside fibers. However, the analyzed vapors flowed in low concentrations (acetic acid and toluene up to 1000 and 1200 ppm, respectively) to avoid the poisoning of the sensing layers. Such a missing effect (i.e., the poisoning) was supported by no change in the baseline of the transient sensor responses, confirming that no chemical interaction had resulted in a permanent variation of the polymer structure. Thus, known concentrations of toluene vapors were generated and flowed throughout the sensor measuring chamber, and the related electrical changes are depicted in Figure 8. The shape of the transient responses (Figure 8a) pointed out quick responses to toluene at each temperature, ranging between 50 and 70 °C, suggesting a Langmuir-like kinetics and reaching the plateau in a few minutes. However, unexpectedly, the sensor response (current values) increased by heating (Figure 8), reporting the highest sensitivity at 70 °C (S_Tol70_: 1.76 × 10^−3^ ppm^−1^; S_Tol50_: 9.63 × 10^−5^ ppm^−1^). The exposure to acetic acid vapors also induced fast responses, linearly related to increasing concentrations of the sample (Figure 9). At higher temperature, sensitivity to acetic acid increased too (S_AcAc70_: 14 × 10^−3^ ppm^−1^; S_AcAc50_: 4.4 × 10^−4^ ppm^−1^) according to an exponential rate (Figure 9d,e). Further, a better signal/noise ratio was gathered as the temperature increased. 

**Figure 10:**
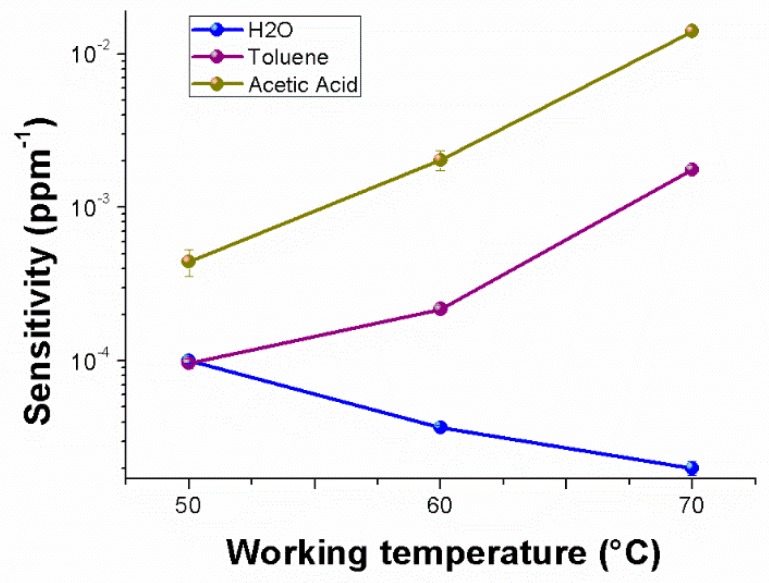
Sensitivity values changes to water vapors, toluene and acid acetic, respectively, depending on the sensor working temperature.

Figure 10 depicts, in semi-logarithm scale, a comparison among the sensitivities to the selected chemicals, enhancing the different affinity of the material to each analyte, the same positive trend for the VOCs and the divergent trend (i.e., sensitivity decreasing) for water vapors. Therefore, going from 50 to 70 °C, the sensitivity to water vapors became five times lower; conversely, to acetic acid and toluene, it was about 32 and 18 times greater, respectively. Another significant sensor feature is the limit of detection (LOD) defined as the lowest concentration of the analyte that can be detected by the sensor under given conditions, particularly at a given temperature. Thus, LOD_AcAc70_ and LOD_Tol70_ (three standard deviations of the blank) were lowered up to ~1 and ~3 ppm, respectively.

**Figure 11:**
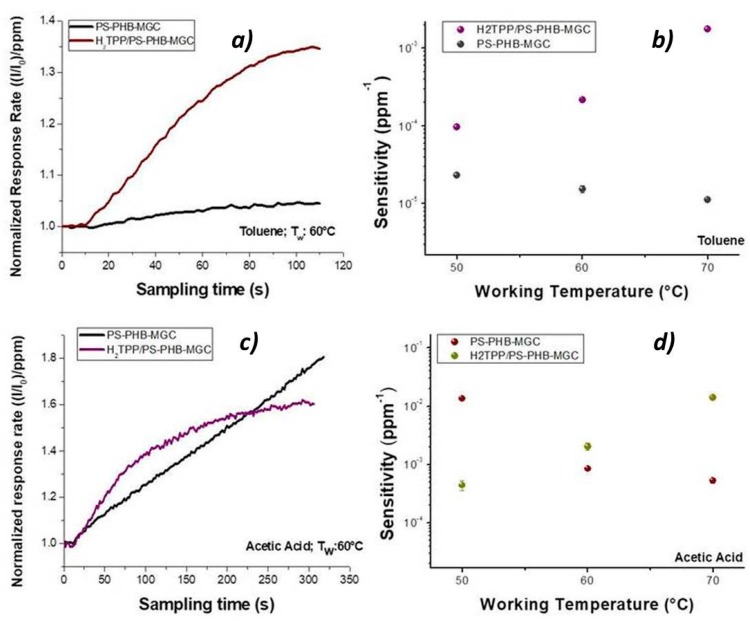
Comparison of the normalized response rate (**a**,**c**) and sensitivities (**b**,**d**) of H_2_TPP-PsB-MGC and PsB-MGC to toluene (**a**,**b**) and acetic acid (**c**,**d**), respectively.

Porphyrin contribution to the sensor features was highlighted by the shape of the normalized response rate, which described the current variation per ppm in time during the exposure to the VOCs flow (Figure 11a). When toluene molecules kept in touch with the fibrous surface within the first 60 s (60 °C), a seven times higher response rate to toluene was measured for H_2_TPP-PsB-MGC sensor than PsB-MGC (specifically from 6.75 × 10^−4^ ± 1.95018 × 10^−5^ ppm^−1^ s^−1^ to 4.90 × 10^−3^ ± 6.27 × 10^−5^ ppm^−1 ^s^−1^). This temperature value was chosen because the current values of both sensors were comparable (Figure 11). The kinetic adsorption profile followed a classical Langmuir profile in both sensors but with different magnitude and adsorption rate. Since both sensors were operating at the same temperature and same toluene concentration, the main parameters involved in the sensor response were expected to be related to the number of the available binding sites and the adsorption energy (features defining the affinity between analyte and surface) [66]. Additionally, toluene could efficiently mediate the electron transfer between porphyrin and MGC. On the other hands, this organic compound could provide conformational changes of both macromolecules and hosting polymer chains (PS), thus contributing to the redistribution of the graphene network, which is responsible for the charge flow. When the sensors were exposed to acetic acid, the H_2_TPP inside fibers apparently was responsible for the changes in both response magnitude and adsorption curve shape, Langmuir-like and Henry-like to with and without porphyrin sensor, respectively (Figure 11), indicating a higher affinity of porphyrin fibers to the analyte. Both transient response and calibration curves are the results of the ad/absorbing processes that depend on the chemical affinity of the VOCs to the material. The nanofibrous thin film can be considered as a complex and heterogeneous system where MGCs, and their arrangement through the fibers, are responsible for the resulting electrical features. Thus, the analytes have to be able to diffuse through the polymer–porphyrin matrix to be adsorbed onto the mesopores (or structural defects) and/or the planar surfaces of graphene, determining the changes in the charge density. The mesoporous structure could work as nucleation center for entrapping and growing molecules, such as AcAc, with multiple functional groups. Simultaneously, specific hydrogen bond interaction with the four nitrogen atoms, arranged in the core of the macrocycle structure, could occur [67]. Since all VOCs induced a rise in current, the effect on network distribution inside fibers could be considered the dominant one. The active contribution of porphyrin to the VOCs adsorption and to the electrical mechanisms is visualized in Figure 11b,d, whereas the changes of sensitivities in temperature for both sensors enhanced the opposite curve trend: sensitivity decreasing by heating in PsB-MGC sensor and conversely increasing in H_2_TPP-PsB-MGC. 

## 4. Conclusions

The present study reported the development of a conductive nanofibrous and nanocomposite polymer sensor combined with a free-base tetraphenylporphyrin, having the role of driving the selectivity and sensitivity of the polymer layer. Electrospinning technology allowed, in a single step and for 2 min, the fabrication of a pink-colored and highly porous layer adhering to the surface of the electrode. The sensor was able to work in a stable and reproducible way between 50 and 70 °C without any significant degradation, and revealing non-linear relationships between the conductivity and the temperature. The electrical conductivity increased when temperature increased, presumably due to the improving of the connectivity of the MGC networks. The effects of the porphyrin appeared significantly in the morphology of fibers (which were smoother and thinner than the porphyrin-free fibers) and in the electrical features. In fact, H_2_TPP-Ps-MGC resulted more resistive at lower temperature, but became much more conductive than PsB-MGC when the chemiresistor worked at 60–70 °C, due to the rearrangement of MGC through the polymer fiber and presumably favored by the aromatic planes of H_2_TPP facing graphene surfaces and phenyl groups of PS. It means that, at a higher temperature, H_2_TPP tends to promote the fibrous layer conductivity. Furthermore, porphyrin not only increased the sensor sensitivity to toluene vapor (i.e., adsorption and diffusion favored), which was not revealed by PsB-MGC, but also increased with increasing temperature, differently from what occurred in PsB-MGC, whereas the sensitivity to VOCs decreased with heating. Further studies are needed to understand the whole mechanism of ad/absorption occurring between MGC–polymer–porphyrin and the VOCs/gas as well as the role of each polymer inside the fibers when the working temperature changed. However, this preliminary study suggests that this complex and nanostructured polymer matrix is expected as a challenging tool, where sensitivity and selectivity, now driven by temperature and a free-base porphyrin, would be designed and ruled taking into account a series of new combinations to create polymer composite sensors able to work alone or in array, at low cost, with fast responses, easy to be produced in large-scale and to be applied for multifaceted environments.
